# Soziale Teilhabe und Hautkrebs während der COVID-19-Pandemie

**DOI:** 10.1007/s00105-022-05093-3

**Published:** 2023-01-16

**Authors:** Jacqueline Niewolik, Marie Mikuteit, Dominik Schröder, Stephanie Heinemann, Gloria Heesen, Frank Müller, Alexandra Dopfer-Jablonka, Imke Grimmelmann, Sandra Steffens

**Affiliations:** 1 Klinik für Rheumatologie und Immunologie, Medizinische Hochschule Hannover, Carl-Neuberg-Str. 1, 30625 Hannover, Deutschland; 2 Lehr- und Lernforschung, Medizinische Hochschule Hannover, Hannover, Deutschland; 3 Institut für Allgemeinmedizin, Universitätsmedizin Göttingen, Göttingen, Deutschland; 4 Deutsches Zentrum für Infektionsforschung Standort Hannover-Braunschweig, Braunschweig, Deutschland; 5 Klinik für Dermatologie, Allergologie und Venerologie (Haut-Tumor-Zentrum), Medizinische Hochschule Hannover, Hannover, Deutschland

**Keywords:** SARS-CoV‑2, Melanom, Immuncheckpointinhibitoren, Social Distancing, Soziale Isolation, SARS-CoV‑2, Melanoma, Immune checkpoint inhibitors, Social distancing, Social isolation

## Abstract

**Hintergrund:**

Die getroffenen Maßnahmen zur Eindämmung der Ausbreitung der Coronavirus-2019-Erkrankung (COVID-19) schränken die sozialen Teilhabemöglichkeiten vieler Menschen ein. Insbesondere Menschen mit chronischen Erkrankungen waren hiervon betroffen. Ziel dieser Beobachtungsstudie war die Untersuchung der sozialen Teilhabe bei Patient:innen mit fortgeschrittenem malignem Melanom mit Immuntherapie unter Pandemiebedingungen. Damit wurde erstmalig soziale Teilhabe als Endpunkt in einer sehr spezifischen Gruppe untersucht. Dies kann als Basis für folgende Studien im wachsenden Kollektiv von Tumorlangzeitüberlebenden in Pandemiezeiten verstanden werden.

**Methodik:**

Querschnittuntersuchung von Melanompatient:innen mit Erhebung des Index zur Messung von Einschränkungen der Teilhabe (IMET). Ergebnisse werden mit publizierten Normdaten verglichen.

**Ergebnisse:**

Es wurden 47 Patient:innen mit malignem Melanom in der Auswertung berücksichtigt. Sie waren im Mittel 58,5 Jahre (SD 13,2) alt, 18 Patient:innen befanden sich im Stadium III und erhielten eine adjuvante Immuntherapie; 29 Patient:innen wurden wegen eines metastasierten Melanoms (Stadium IV) behandelt. Die Ergebnisse des IMET ergaben im Gesamtscore keine signifikanten Einschränkungen der sozialen Teilhabe im Vergleich zu den publizierten vorpandemischen Normdaten. Teilnehmerinnen hatten jedoch eine signifikant eingeschränktere Teilhabe.

**Diskussion:**

Der Endpunkte soziale Teilhabe rückt bei Langzeitüberlebenden mit malignem Melanom in den Vordergrund. Unter Pandemiebedingungen sind insbesondere Patientinnen von Einschränkungen der sozialen Teilhabe gefährdet. Eine Differenzierung zwischen Pandemieeinflüssen und erkrankungsspezifischen Faktoren geht aus unseren Daten nicht hervor. Ausgehend von dieser Studie können und sollten weitere Erhebungen zur sozialen Teilhabe in Zeiten von pandemischen Infektionserkrankungen, insbesondere im wachsenden Kollektiv onkologischer Langzeitüberlebender erfolgen.

**Graphic abstract:**

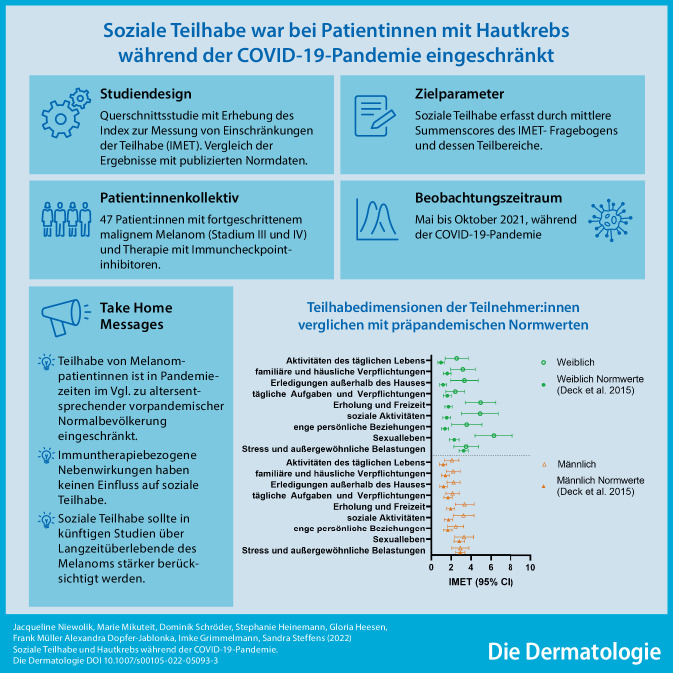

## Abstract (en)

**Background:**

Measures used to contain the spread of coronavirus disease 2019 (COVID-19) have impaired the social participation of many people. Especially people with chronic or oncologic conditions were affected. In this observational study, we aimed to assess social participation in melanoma patients with immune checkpoint therapy. Therefore, we investigated social participation in a very specific group and consider this a basis for further studies in a growing collective of long-term survivors in an era of infectious diseases.

**Materials and methods:**

Cross-sectional assessment via the index to measure social participation and health associated quality of life (“Index zur Messung von Einschränkungen der Teilhabe”, IMET). Results are compared with published norm data.

**Results:**

In all, 47 melanoma patients (mean age 58.5 years, standard deviation 13.2) were included: 18 patients had advanced melanoma stage III and received adjuvant Immunotherapy, while 29 patients were treated because of metastatic melanoma (stage IV). Results of the IMET showed no significant impairments in social participation when regarding the total score and when being compared to prepandemic norm data. Female patients did have significantly impaired participation.

**Conclusion:**

The endpoints health-related quality of life and social participation are important in long-term survivors with melanoma. During the pandemic, women of our collective were especially at risk for impairment of social participation. Differences between pandemic and disease-specific influences on social participation cannot be derived from our data. Nevertheless, this study will be part of growing and necessary research on long-term cancer survivors diseases.

Die Ausbreitung der Coronavirus-Erkrankung 2019 (COVID-19) zur globalen Pandemie und die zur Eindämmung getroffenen Maßnahmen gingen mit Einschränkungen des öffentlichen und privaten Lebens einher. Diese Einschränkungen haben unmittelbare Auswirkungen auf die Teilhabe am gesellschaftlichen Leben. Wir berichten über die soziale Teilhabe bei Melanompatient:innen unter Immuntherapie während der COVID-19-Pandemie.

Die Einführung der Immuntherapie mit Checkpointinhibitoren (ICI) verbesserte die Prognose von Melanompatient:innen in fortgeschrittenen Erkrankungsstadien in den letzten Jahren drastisch [9, 20, 21]. Angesichts längerer Behandlungsverläufe wird das psychosoziale Wohlergehen als langfristiges Behandlungsziel zunehmend wichtiger. Vor der COVID-19-Pandemie durchgeführte Erhebungen bei Patient:innen mit fortgeschrittenem malignen Melanom unter oder nach Immuntherapie zeigten meist Einschränkungen der Lebensqualität [2, 17].

Gleichzeitig ist die soziale Teilhabe während der Pandemie etwa bei Menschen mit chronisch entzündlichen, rheumatischen oder psychiatrischen Erkrankungen im Vergleich zur Normalbevölkerung stärker eingeschränkt [10, 14].

Soziale Teilhabe ist als patient:innenzentriertes Outcome v. a. in der Rehabilitationswissenschaft weit verbreitet, wird zunehmend jedoch auch bei der Untersuchung onkologischer Patient:innenkollektive angewandt [4, 18, 19]. Die soziale Teilhabe berücksichtigt die Ausgestaltung des Lebens im individuellen sozialen Umfeld und der Gesellschaft und geht somit über individuumzentrierte Ansätze wie die Lebensqualität hinaus.

Ziel unserer Studie ist die Untersuchung von sozialer Teilhabe von Menschen mit fortgeschrittenem malignem Melanom (Stadium III und IV nach AJCC 2018) unter oder nach Immuntherapie während der COVID-19-Pandemie.

## Methodik

### Studiendesign

Die vorliegende Studie basiert auf Daten der nichtinterventionellen Längsschnittstudie CoCo Immun, bei der die Immunantwort, soziale Teilhabe sowie Impfeinstellung von immungeschwächten Menschen untersucht wurden. Das Studienprotokoll beschreibt das genaue Procedere [8]. Als Teilstichprobe von Mai bis Oktober 2021 wurde auch eine Gruppe an Melanompatient:innen in Stadium III oder IV (AJCC Melanoma Staging System [12]) unter Immuncheckpointinhibitoren rekrutiert.

Die Rekrutierung erfolgte im Hauttumorzentrum der Medizinischen Hochschule Hannover (MHH).

Einschlusskriterien waren (a) ein histologisch bestätigtes malignes Melanom (Stadium III oder IV), (b) eine aktive oder frühere Behandlung mit den Immuncheckpointinhibitoren Nivolumab, Pembrolizumab oder Ipilimumab oder einer Kombinationstherapie dieser Medikamente, (c) Alter über 18 Jahre. Von allen Teilnehmenden wurde vor Studienteilnahme eine schriftliche Einverständniserklärung eingeholt.

Für die vorliegende Untersuchung besteht ein positives Votum der Ethikkommission der Medizinischen Hochschule Hannover (9948 BO K 2021). Die übergeordnete Studie wurde im Deutschen Register klinischer Studien registriert (DRKS00023972).

### Fragebögen und Indizes

Der handschriftlich auszufüllende Fragebogen beinhaltete neben soziodemografischen Daten mehrere Indizes und Scores zur Erhebung von pandemiebedingten Einschränkungen und psychischer Gesundheit. Zur Soziodemografie wurden unter anderem Alter und Gender (selbst zugeordnetes, soziales Geschlecht) erhoben, s. auch Tab. 1.

**Table 1:** 

		Gender		
		Weiblich *n* = 18	Männlich *n* = 29	
		*n* (%)	*n* (%)	*p*^a^
*Soziodemografische Parameter*				
Alter	Mittelwert (SD)	59 (15)	59 (13)	0,956
	< 50 Jahre	4 (22,2)	5 (17,2)	0,914
	50–70 Jahre	10 (55,6)	17 (58,6)	
	> 70 Jahre	4 (22,2)	7 (24,1)	
Schulabschluss^b^	Volksschulabschluss/keiner	1 (5,9)	0 (0)	0,136
	Hauptschulabschluss	1 (5,9)	3 (12,5)	
	Mittlere Reife	8 (47,1)	5 (20,8)	
	(Fach‑)Hochschulreife	7 (41,2)	16 (66,7)	
Arbeitszeit^c^	Teilzeit < 15 h/Woche	3 (37,5)	0 (0)	0,057
	Teilzeit 15–34 h/Woche	1 (12,5)	2 (15,4)	
	Vollzeit > 35 h/Woche	4 (50)	11 (84,6)	
Rente^d^	Altersrente/Pension	6 (35,3)	8 (30,8)	0,757
	Erwerbsunfähigkeitsrente	3 (17,6)	1 (3,8)	0,284
Migrationshintergrund^e^		0 (0)	2 (9,1)	0,495
*Klinische Parameter*				
Tumorstadium	IIIa–d	6 (33,3)	12 (41,4)	0,581
	IV	12 (66,7)	17 (58,6)	
Letzte ICI-Therapie	Monate Mittelwert (SD)	15 (16)	14 (13)	0,379
	≤ 6 Monate	9 (50)	11 (37,9)	0,577
	> 6 Monate	9 (50)	18 (62,1)	
„Immune-related adverse events“^f^		15 (83,3)	19 (65,5)	0,315
Therapieregime	Monotherapie PD-1-Inhibitor	11 (61,1)	20 (69,0)	0,570
	Monotherapie Ipilimumab	2 (11,1)	1 (3,4)	
	PD-1-Inhibitor + Ipilimumab	5 (27,8)	8 (27,6)	

*SD* Standardabweichung

^a^ Chi-Quadrat/Fisher’s Exact-Test für kategoriale Variablen und Mann-Whitney-*U*-Test für metrische und kategoriale Variablen

^b^ Keine Angabe *n* = 6

^c^ Keine Angabe/nicht zutreffend *n* = 26

^d^ Keine Angabe *n* = 4

^e^ Keine Angabe *n* = 8, Migrationshintergrund nach der Definition der Agentur für Arbeit: die Person selbst oder mindestens eins ihrer Elternteile ist nach 1949 nach Deutschland zugewandert

^f^ Immunbezogene unerwünschte Nebenwirkungen, definiert durch die „National Cancer Institute Common Terminology Criteria“ (CTCAE)

Der Index zur Messung von Einschränkungen der Teilhabe (IMET [6]) umfasst 9 Items, die die soziale Teilhabe in verschiedenen Dimensionen erfassen. Die abgefragten Items fokussieren übliche Aktivitäten des täglichen Lebens, familiäre und häusliche Verpflichtungen, Erledigungen außerhalb des Hauses, tägliche Aufgaben und Verpflichtungen, Erholung und Freizeit, soziale Aktivitäten, enge persönliche Beziehungen, Sexualleben und Stress und außergewöhnliche Belastungen. Für jedes Item werden im IMET 11 Level auf einer Likert-Skala von 0 (keine Einschränkungen) bis 10 (größtmögliche Einschränkung) erhoben. Die Summe der Items zeigt die Einschränkungen der sozialen Teilhabe mit hoher interner Reliabilität (Cronbachs Alpha = 0,90) [5]. Wir vergleichen die Daten unserer Kohorte mit vorpandemischen Normdaten von Deck et al. in der Altersgruppe 50 bis 59 Jahre [7]. Die von Deck et al. als Grundlage für die Normdaten gewählte Stichprobe umfasst *n* = 5004 über das Einwohnermeldeamt der Hansestadt Lübeck zufällig gezogene Personen. Das Durchschnittsalter der im Sommer 2014 gezogenen Stichprobe betrug 49,4 Jahre, 50 % der Stichprobe waren weiblich [7].

### Statistische Auswertung

Die Fragebögen wurden mithilfe des EvaSys-Umfragesystems (EvaSys GmbH, Lüneburg, Deutschland) erstellt. Nach der Digitalisierung und dem Einlesen der Fragebögen sowie der Korrektur von automatisch erkannten Fehlern erfolgte ein direkter Export der Daten nach SPSS (Version 27, IBM Corp., Armonk, NY, USA) zur weiteren statistischen Auswertung.

Bei der Berechnung der IMET-Summenscores wurden bis zu 2 fehlende Werte interpoliert (Mittelwert der von den Teilnehmer:innen angegebenen Werte). Teilnehmer:innen mit 3 oder mehr fehlenden Werten beim IMET-Fragebogen wurden von der Auswertung ausgeschlossen.

Metrische Daten wurden als Mittelwerte mit Standardabweichung (SD) oder 95 %-Konfidenzintervallen (CI) angegeben. Die Teststatistik zwischen kategorialen Variablen umfasste den Chi-Quadrat-Test bzw. den Fisher’s Exact- oder Fisher-Freeman-Halton Exact-Test bei erwarteten Zellenwerten von < 5. Bei der teststatistischen Auswertung des Einflusses vom Geschlecht auf die Punktescores fand der Mann-Whitney-U-Test Anwendung. Der lineare Zusammenhang von 2 metrischen bzw. ordinalen Variablen wurde mittels bivariater Rangkorrelation unter Angabe des Korrelationskoeffizienten (r) nach Spearman bemessen. Ein Korrelationskoeffizient von 0,1 deutet nach Cohen (1988) auf eine schwache, 0,3 auf eine moderate und 0,5 auf eine starke Korrelation hin [3]. *p*-Werte unter 0,05 wurden als statistisch signifikant angesehen. Abbildungen wurden mit GraphPad Prism 9.0 (GraphPad Software, San Diego, CA, USA) erstellt.

## Ergebnisse

### Studienpopulation

Im Zeitraum von Mai bis Oktober 2021 wurden 49 Melanompatient:innen für unsere Studie rekrutiert. Davon wurden 2 Patient:innen von der weiteren Auswertung aufgrund fehlender Beantwortung von mehr als 2 Items des Teilhabefragebogens ausgeschlossen.

Der Altersmittelwert lag bei 58,5 Jahren (SD 13,2), 39,5 % der Patient:innen waren weiblich. Der überwiegende Teil der Teilnehmer:innen hatte die ICI-Therapie bereits vor über 6 Monaten vor Studieneinschluss abgeschlossen (57,4 %). Weitere Daten zu Demografie, Tumorerkrankung und -therapie finden sich in Tab. 1.

### Soziale Teilhabe

**Figure 1:**
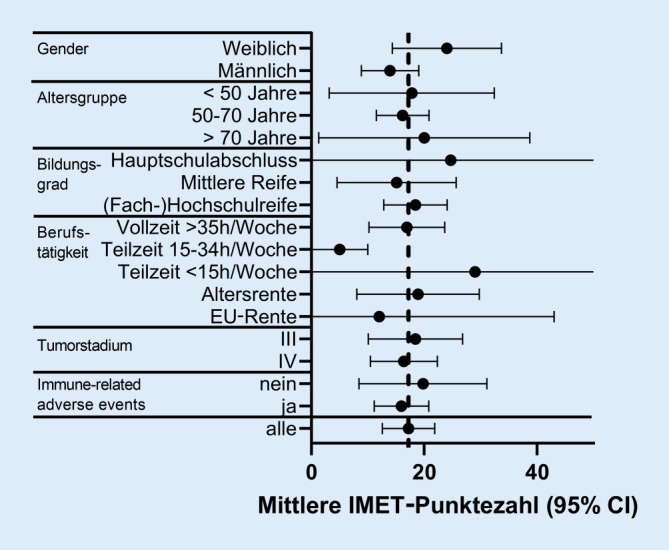

Der Mittelwert des IMET-Summenscores, der die Einschränkung der Teilhabe misst, lag in unserer Kohorte bei 19,2 (SD 16,6). Deutlich über dem Mittelwert liegende, jedoch nicht signifikant erhöhte Summenscores fanden sich bei Teilnehmerinnen, Teilnehmer:innen älter als 70 Jahren oder mit Hauptschulabschluss sowie geringfügig (< 15 h/Woche) Beschäftigten (vgl. Abb. 1).

### Vergleich der sozialen Teilhabe mit Normwerten

**Figure 2:**
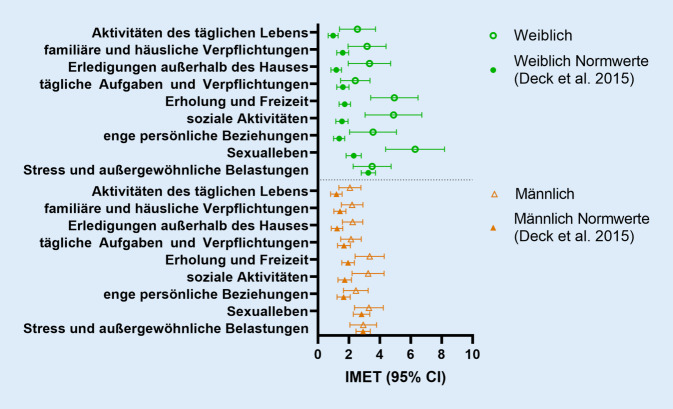

Verglichen mit vorpandemischen Normwerten der Altersgruppe zwischen 50 und 59 Jahren zeigte sich jedoch in einer nach Geschlechtern aufgeteilten Auswertung ein signifikant schlechterer IMET-Score unter den Teilnehmerinnen mit Melanom (25,7 vs. 15,59 Punkte, *p* = 0,04), allerdings kein Unterschied zwischen den Teilnehmern (15,1 vs. 16,6 Punkte, *p* = 0,688). Dieser Unterschied bei den Teilnehmerinnen betrifft v. a. die Bereiche „übliche Aktivitäten des täglichen Lebens“, „Erledigungen außerhalb des Hauses“, „Erholung und Freizeit“, „soziale Aktivitäten“, „enge persönliche Beziehungen“ und „Sexualleben“. Bei Männern zeigte sich lediglich ein grenzwertig signifikanter Unterschied in den Dimensionen „Erholung und Freizeit“ sowie „soziale Aktivitäten“ (vgl. Abb. 2).

## Diskussion

In unserer rekrutierten Kohorte von Melanompatient:innen mit ICI-Therapie zeigten sich bezogen auf die meisten soziodemografischen und krankheitsspezifischen Faktoren keine größere Einschränkungen der sozialen Teilhabe unter Bedingungen der COVID-19-Pandemie. Deutliche und signifikante Unterschiede zu publizierten Normdaten einer vergleichbaren Altersgruppe zeigten sich in der Teilhabe von Studienteilnehmerinnen [7]. Eine geschlechtsbezogene Stichprobenverzerrung erscheint insgesamt unwahrscheinlich, da es für soziodemografische und krankheitsspezifische Faktoren keine signifikanten Unterschiede gibt.

Einschränkungen der Teilhabe konnten während der Pandemie für die Allgemeinbevölkerung und insbesondere auch bei Menschen mit anderen chronischen Erkrankungen reproduziert werden [10, 14]. Im Vordergrund stehen dabei, wie auch in unserer Studie, Einschränkungen der Teilbereiche „soziale Aktivitäten“ und „Erholung und Freizeit“ unabhängig von Geschlecht. Dies interpretieren wir im Rahmen der allgemeinen Kontaktbeschränkungen und Social-Distancing-Maßnahmen der COVID-19-Pandemie. Die insbesondere bei Patientinnen eingeschränkte Teilhabe im Sexualleben, in engen persönlichen Beziehungen und Erledigungen außerhalb des Hauses können auf eine z. B. durch Homeoffice und Homeschooling bedingte vermehrte Belastung durch Sorgearbeit in der Familie und in Partner:innenschaften zurückzuführen sein. Eine israelische Studie zeigte eine Assoziation von weiblichem Gender mit mehr Distress-Leveln und geringerer Lebensqualität während der COVID-19-Pandemie [11]. Interessant ist im Zusammenhang von sozialer Teilhabe und COVID-19-Pandemie auch die individuelle Risikoeinschätzung. Bei erwartetem höherem Risiko durch eine mögliche COVID-19-Erkrankung ist ggf. auch eine größere Einschränkung der sozialen Teilhabe durch sozialen Rückzug zu erwarten. Laut Studienlage haben Melanompatient:innen mit ICI-Therapie kein erhöhtes Risiko für einen schweren COVID-19-Verlauf oder auch ein verschlechtertes Impfansprechen [15, 16]. Die individuelle Risikowahrnehmung kann davon selbstredend unbeeinflusst bleiben. Alsharawy et al. zeigten beispielsweise eine höhere Risikoerwartung bei Frauen [1]. Dies könnte den von uns gezeigten genderassoziierten Unterschied mitbedingen.

Als Grund für die eingeschränkte Lebensqualität von Melanompatient:innen unter ICI-Therapie im Allgemeinen sollte auch der Einfluss von immunassoziierten Nebenwirkungen (irAE) diskutiert werden. Mamoor et al. fanden in ihrer Untersuchung von Melanompatient:innen nach ICI-Behandlung eine gute Lebensqualität, aber hohe Raten an Fatigue und muskuloskeletalen Beschwerden [13]. Auch in unserer Kohorte litten 72,3 % der Teilnehmenden an irAE, wir konnten jedoch keine Assoziation mit verminderter sozialer Teilhabe feststellen.

Trotz zunehmender Relevanz von sozialer Teilhabe unter prognoseverbessernden Therapien wie den ICI ist diese in der Literatur – auch vor der COVID-19-Pandemie – bei Melanompatient:innen noch zu wenig untersucht.

### Stärken und Limitationen

Eine klare Limitation unserer Untersuchung ist die geringe Fallzahl. Grundlage der Untersuchung war zudem ein Vergleich mit den von Deck et al. publizierten Normdaten in einer präpandemisch erhobenen (2014) zufällig gezogenen Stichprobe aus Norddeutschland [7]. Dementsprechend ist aus unseren Daten keine Differenzierung des Pandemieeinflusses vs. erkrankungsspezifischer Faktoren möglich. Unsere Studie ist nach unserem Kenntnisstand jedoch die erste, welche explizit soziale Teilhabe in diesem speziellen Patient:innenkollektiv und unter Pandemiebedingungen beschreibt. Damit bietet sie einen Ausgangspunkt für weitere notwendige Untersuchungen im wachsenden Kollektiv Tumorlangzeitüberlebender in Zeiten von sich weiter ausbreitenden Infektionserkrankungen.

## Fazit für die Praxis

- Melanompatientinnen unter Immuncheckpointinhibitor-Therapie haben während der Pandemie eine eingeschränktere Teilhabe im Vergleich zur altersentsprechenden präpandemischen Normalbevölkerung.
- Immuntherapiebezogene Nebenwirkungen der Immuncheckpointinhibitoren haben keinen Einfluss auf die soziale Teilhabe.
- Soziale Teilhabe ist eine wichtige Kenngröße bei Langzeitüberlebenden des malignen Melanoms und sollte in künftigen Studien stärkere Berücksichtigung finden.
